# Astrocyte properties in cetacean cortices

**DOI:** 10.14814/phy2.70651

**Published:** 2025-11-10

**Authors:** Anu Venkatesh, Abby M. McClain, Carolina R. Le‐Bert, Whitney B. Musser, Sam H. Ridgway

**Affiliations:** ^1^ Naval Information Warfare Center Pacific San Diego California USA; ^2^ National Marine Mammal Foundation San Diego California USA; ^3^ Department of Pathology University of California San Diego California USA

**Keywords:** astrocytes, cetaceans, cortical glia, marine mammal

## Abstract

Cetacean neurons are far more extensively studied in the scientific literature than the other principal cell type of the central nervous system—glia. To help address this knowledge gap, the current study profiled astrocytes in five cetacean species—*Tursiops truncatus* (*Tt*), *Orcinus orca* (*Oo*), *Ziphius cavirostris* (*Zc*), *Pseudorca crassidens* (*Pc*), and *Kogia breviceps* (*Kb*) with brain masses ranging from 596 g in *Kb* to 6215 g for *Oo*. Using formalin‐fixed brain tissues stained with anti‐glial fibrillary acidic protein antibodies, astrocyte distributions across cortical regions were profiled for each animal, including measurements of astrocyte diameter. Results showed statistically significant (*p* < 0.003) effects for cortical layer and species in addition to astrocyte size (cell body) between the animals. The largest astrocytes were found in the larger cetaceans (*Pc*), although average astrocyte size did not statistically differ from the control (*Mus musculus*). The results of this investigation advance our knowledge of cetacean astrocyte biology, with translational implications for human conditions such as dementia and traumatic brain injury. Collectively, these findings illustrate a quantitative approach to better understand neuroanatomical variations across cetacean species.

## 1 INTRODUCTION

While cetacean neurons are extensively studied, relatively little is known regarding the non‐neuronal cells of the central nervous system. Glial cells, a major non‐neuronal cell type in the brain, comprise nearly 50% of human brains, with percentages greater than 70% reported in one species of large cetacean, the minke whale (*Balaenoptera acutorostrata*; Verkhratsky & Nedergaard, 2016). Given that glia maintain the cellular architecture of the brain in addition to regulating neurotransmitters, ion gradients, gas exchange, nutrients, and pathogens (Lundgaard et al., 2014), it is important to characterize this cell type across different mammalian species. One subtype of glia, the astrocyte, was first profiled in cetaceans over 30 years ago (Pritz‐Hohmeier et al., 1994), with only a handful of papers published on the topic since. Recent work from our group has documented neuron and astrocyte densities across five odontocete species (*Tursiops truncatus*, *Delphinus delphis*, *Orcinus orca*, *Ziphius cavirostris*, and *Kogia breviceps*) and proposed that neuronal density may be inversely related to dive time (Ridgway et al., 2019). However, rigorous statistical comparisons were not made between species nor were detailed measurements of astrocytes performed.

Previous studies of cetacean brains focused on the laminar distributions of neurons and glia in the cortex. Hof et al. (2005) documented a prominent, but neuron‐sparse layer I (L1) in the bottlenose dolphin (*T. truncatus*) neocortex and variation in neuron densities across layers II, III, and VI (L2, L3, L6; 2005). In contrast, layer IV (L4) was not mentioned and is thought to be difficult to discriminate or is absent in cetaceans (Kesarev & Malofeeva, 1969; Ridgway et al., 2019). Similar findings were reported across four additional reports in cetacean brains (Cozzi et al., 2016; Garey & Leuba, 1986; Kesarev & Malofeeva, 1969; Spocter et al., 2017). Of these, both Spocter et al. (2017) and Garey and Leuba (1986) note that lower densities of neurons are accompanied by higher glial numbers, with Garey suggesting that these differences were not due to the animal's age nor brain region. None of the previous studies, however, examined the morphology and distribution of the two types of astrocytes—fibrous and protoplasmic. Fibrous astrocytes are typically found in white matter (WM), while protoplasmic astrocytes are found in gray matter. Each type differs in both function, form, and distribution (Macnab & Pow, 2007; Oberheim et al., 2012). For example, WM astrocytes have far fewer radiating processes and rely less on gap junctions for rapid calcium signaling and glutamate uptake compared to gray matter astrocytes. Thus, the present study includes cortical WM as a layer of interest in addition to L1–L5 in gray matter.

Beyond layer specific characteristics, it is presently unknown if the size of fibrous astrocytes in cetaceans differs from astrocytes in other mammals, such as humans or rodents. Challenges exist for accurate size profiling since both the cell body and its processes must be aligned with the appropriate plane of imaging (Falcone et al., 2019). In humans, measurements of protoplasmic astrocytes demonstrate that human astrocytes have a 2.6‐fold larger diameter than rodent astrocytes (Oberheim et al., 2009). Presumably, this difference in size may contribute to species‐specific differences in intelligence since larger astrocytes can regulate more synapses and thus, more effectively regulate ion gradients and neurotransmitters (Blackburn et al., 2009; Vasile et al., 2017). Since fibrous astrocytes have fewer processes than the protoplasmic subtype (Oberheim et al., 2012), measurements of soma diameter are feasible in WM using standard fluorescent microscopy. This is a first step to documenting potential morphologic differences among mammalian brain tissue.

The present study aims to describe fibrous astrocyte anatomical characteristics in cortical gray and white matter of five cetacean species. Comparisons between species and cortical regions will provide a deeper understanding of the fibrous astrocyte distribution differences between species. Furthermore, the results from this study will provide a platform from which future studies could expand upon the cellular diversity and function of cetacean neural tissue.

## 2 MATERIALS AND METHODS

### 2.1 Animals

Five odontocete brains (*Pseudorca crassidens*, *Kogia breviceps*, *Tursiops truncatus*, *Orcinus orca*, and *Ziphius cavirostris*; abbreviated as *Pc*, *Kb*, *Tt*, *Oo*, and *Zc*, respectively) were removed at necropsy within 12 h of death and placed in 10% neutral buffered formalin prior to examination (Ridgway et al., 2019). All animals had died of natural causes under professional care or after stranding on beaches. The *Zc* and *Kb* stranded on beaches in California and were collected by local stranding networks authorized under the United States Marine Mammal Protection Act. The U.S. Navy Marine Mammal Program (MMP) cared for the *Tt* and *Oo* prior to death. Brains were collected under the authority codified in U.S. Code, Title 10, Section 7524. Secretary of Navy Instruction 3900.41H directs that Navy marine mammals be provided the highest quality of care. The MMP, Naval Information Warfare Center (NIWC) Pacific, currently houses and cares for a population of bottlenose dolphins and California sea lions in San Diego Bay (CA, USA). NIWC Pacific is accredited by AAALAC International and adheres to the national standards of the U.S. Public Health Service policy on the Humane Care and Use of Laboratory Animals and the Animal Welfare Act (Table 1).

**TABLE 1:** Summary of animals, age, sex, and brain size prior to dissection.

Species	Age	Sex	Brain size (g)
Pseudorca crassidens (*Pc*)	Subadult	F	Unknown
Kogia breviceps (*Kb*)	Subadult	F	596
Tursiops truncatus (*Tt*)	Adult	F	1559
Orcinus orca (*Oo*)	Adult	F	6215
Ziphius cavirostris (*Zc*)	Adult	F	2004

### 2.2 Histology

Histological staining, including Luxol Fast Blue (LFB), anti‐glial fibrillary acidic protein (GFAP) marker, and Nissl were completed through Reveal Biosciences (Reveal Biosciences, San Diego, CA, http://www.revealbio.com). Formalin‐fixed, paraffin‐embedded tissue samples were sectioned into 6 μm thick slices. Following this, LFB and Nissl staining were performed according to standard Reveal Biosciences protocol. For LFB staining, a 0.1% LFB MBS Solution (Electron Microscopy Solutions [EMS], Hatfield, PA, cat #26681‐01) was used with 0.05% Lithium Carbonate and 0.1% Cresyl Echt Violet (cat # 26089‐01EMS). For Nissl staining, 2% Cresyl Violet Acetate was used (cat # 26089‐20EMS). For GFAP, immunofluorescence staining was completed on a Leica Bond automated immunostainer (Leica Biosystems, Buffalo Grove, IL, https://www.leicabiosystems.com). A 20‐min heat‐induced antigen retrieval was performed with pH 6.0 Citrate, followed by an incubation of rabbit anti‐GFAP primary (1:2000 dilution; Abcam/ab7260) at 4°C overnight, and a fluorescent conjugated secondary (1:200 dilution; Anti‐Rabbit IgG (H+L) Alexa Fluor Plus 647, Thermofisher A31573) for 60 min at room temperature. A counter stain with DAPI (EMS, cat#17985‐51) and a negative control using no primary antibody was also performed.

### 2.3 Tissue profiling

Due to limited tissue availability, different areas of cortex were profiled for each animal: motor and auditory cortex for *Pc*; somatosensory, parahippocampal and motor cortex for *Kb*; motor cortex for *Tt*; auditory, somatosensory and visual cortex for *Zc*; and motor and auditory cortex for *Oo*. Quantification of astrocytes via GFAP+ staining was completed using Image J software (Schneider et al., 2012), whereas cell diameter was measured using 3DHISTECH's CaseViewer2.0 (https://www.3dhistech.com/solutions/caseviewer/). Using ImageJ, images were converted to a Red‐Green‐Blue (RGB) stack and then manually thresholded using the slider to cover the roughly spherical areas positive for GFAP. Manual thresholding ensured that staining from background sources was limited and consistent across images. During thresholding, the researcher was blinded to the details of tissue source (e.g., region and species). Pixels of positive GFAP staining were converted to a percentage of the total area (5mm^2^) using the protocol from Denver (2016).

Three tissue subsections of 5mm^2^ each were selected for each layer (four total), resulting in a total of 12 sections per animal, per brain region. Layers of cortex were identified by eye using landmarks in GFAP+ and Nissl staining and subdivided into L1, L2/3, L5 and WM by a trained researcher. L1 was identified by examining the most superficial layer marked with a high density of GFAP + cell bodies and low Nissl staining; L2/3 and L5 were identified as a horizontal striation of sparse cell bodies for GFAP but high Nissl staining for neurons, surrounded by areas of limited staining with L5 being deeper in the tissue, lastly WM was marked by the highest LFB staining for myelin. L2 and L3 were very difficult to distinguish for GFAP+ staining in these tissue samples, consistent with previous accounts in cetacean neocortex (Glezer et al., 1992); therefore, L2 was grouped with L3 whereas L4 is thought to be absent in cetacea. Visualization of cortical layers was more difficult than accounts in human tissue (Palomero‐Gallagher & Zilles, 2017), especially for GFAP+ astrocytes.

**FIGURE 1:**
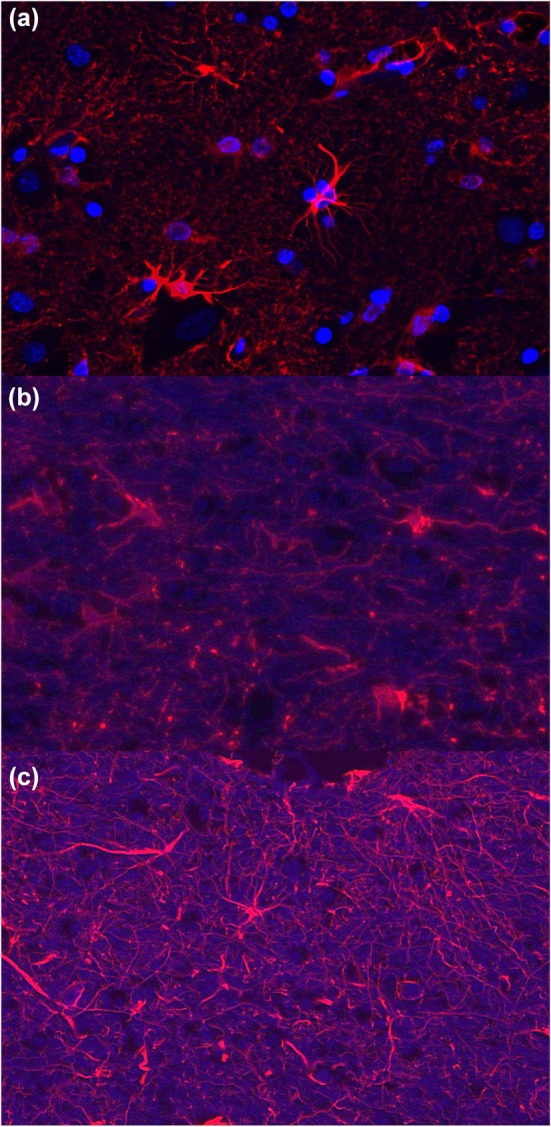
Representative images of white matter astrocytes stained with anti‐GFAP antibodies and counter‐stained with DAPI. (a) motor cortex for *Tursiops truncatus*, (b) visual cortex for *Ziphius cavirostris*, (c) auditory cortex for *Pseudorca crassidens*.

Cell diameters were measured in fibrous astrocytes along the longest plane of the cell body. The inclusion criteria for each cell included (1) uniform GFAP staining, (2) in‐plane orientation of the soma, and (3) expected morphology for fibrous astrocytes (Verkhratsky & Nedergaard, 2016) including less complexity and fewer processes. For each animal, *n* = 12 astrocytes (see Figure 1 for representative examples) were selected and *Mus musculus* (*Mm*) was included for comparison. For some animals, suitable astrocytes for measurement were not available within a single region (e.g., motor cortex) so astrocytes across multiple regions were pooled to reach the desired *n* for rigorous comparison between species.

### 2.4 Statistics

One and two‐way repeated measures ANOVAs were run using GraphPad Prism, Version 9.3.1 for Mac, GraphPad Software, San Diego, California USA, www.graphpad.com. Given the small sample sizes for astrocyte distribution, a Greenhouse–Geisser correction was applied to the two‐way ANOVAs to lower the likelihood of Type‐1 error.

## 3 RESULTS

### 3.1 Distributions by cortical layer

To compare astrocyte distributions in cortex, the percentage of GFAP+ staining was compared across layers. Although tissue sections were collected across different brain regions, this analysis was limited to motor cortex in *Pc*, *Kb*, *Tt*, and *Oo*. A two‐way Species × Layer repeated measures ANOVA revealed a significant effect of Species, F (3, 8) = 11.37, *p* < 0.003 with *Tt* having the highest percentage of GFAP staining (Mean ± SD; 3.82 ± 1.87%), followed by *Kb* (2.64 ± 1.54%), *Oo* (2.45 ± 2.39%), and *Pc* (1.85 ± 1.37%). A significant effect of Layer was also observed, F (1.83, 14.65) = 94.96, *p* < 0.001, with L1 having the most staining (4.17 ± 0.77%), followed by WM (4.06 ± 2.12%), L2/3 (1.66 ± 1.02%), and L5 (0.87 ± 0.62%). Lastly, a significant Species × Layer interaction was also observed, *F* (9, 24) = 10.23, *p* < 0.001.

**FIGURE 2:**
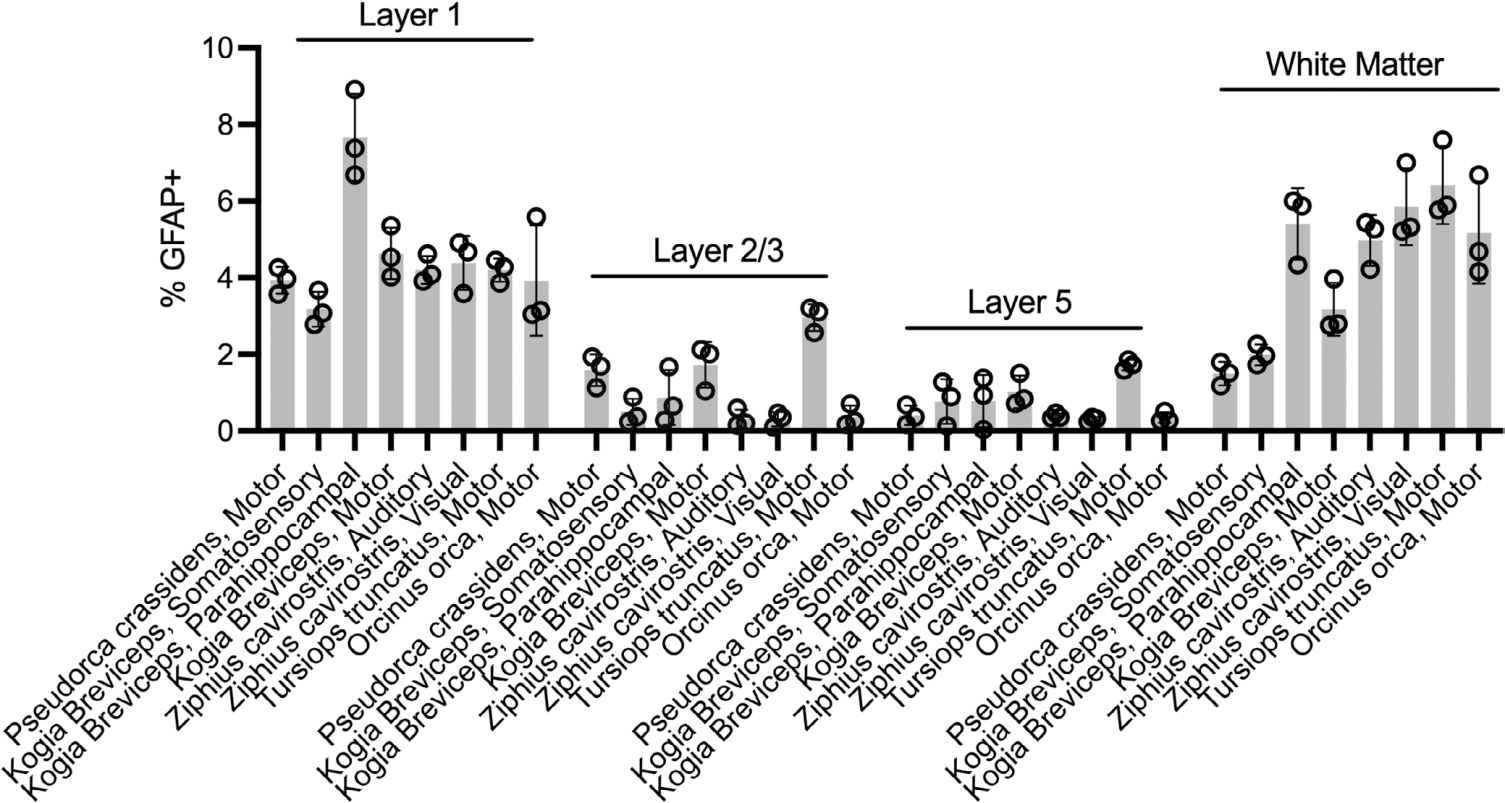
Percentage of astrocytes with anti‐glial fibrillary acidic protein (GFAP+) pixels per tissue subsection. Multiple subsections were taken for each layer and brain region.

The pattern of higher GFAP staining in L1/WM and lower staining in L2‐5 was also observed in other brain regions (e.g., somatosensory, parahippocampal, auditory cortices; see Figure 2).

### 3.2 Astrocyte size

**FIGURE 3:**
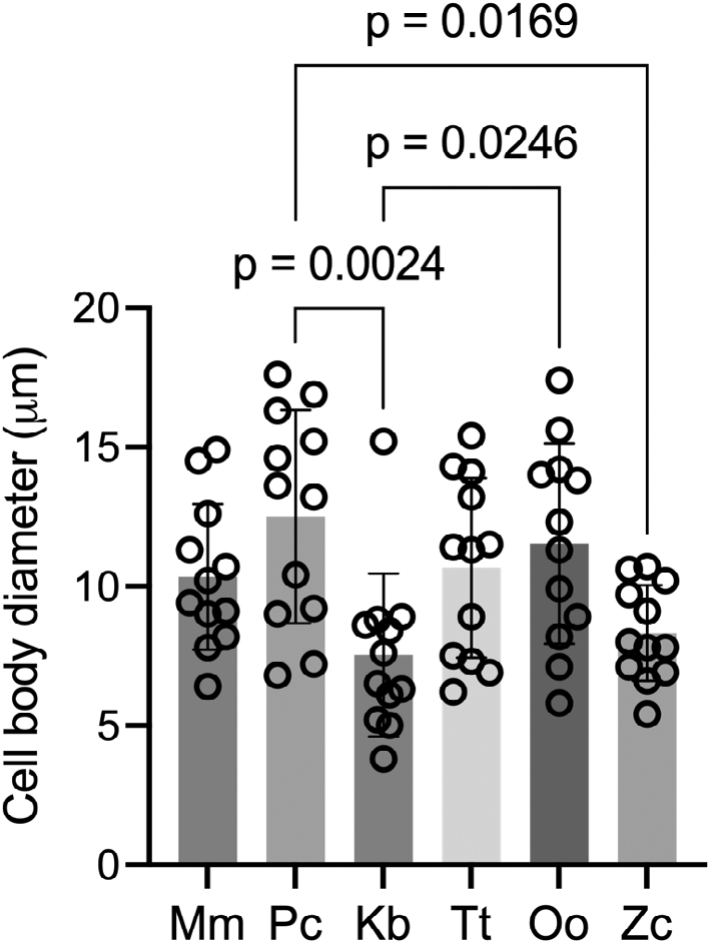
Cell body diameter of astrocytes in the white matter. Multiple, separate cells were measured for each animal. Brain regions were pooled for each animal, significant differences shown. Kb, *Kogia breviceps*; Mm, *Mus musculus*; Pc, *Pseudorca crassidens*; Tt, *Tursiops truncatus*; Oo, *Orcinus orca*; Zc, *Ziphius cavirostris*.

A one‐way repeated measures ANOVA comparing astrocyte diameters revealed a significant effect of species on fibrous astrocyte size, *F* (6, 66) = 10.78, *p* < 0.01. Post‐hoc Tukey's multiple comparisons revealed that astrocyte diameter (μm) in *Pc* (average ± standard deviation; 12.50 ± 3.83) was significantly (*p* < 0.05) larger than in *Zc* (8.33 ± 1.72) and *Kb* (7.53 ± 2.93), and mean diameters for *Kb* were significantly smaller than *Oo* (11.54 ± 3.61). However, the mean astrocyte diameter in *Mm* (10.34 ± 2.61) did not differ from any of the marine mammals (*p*'s > 0.51), see Figure 3.

To account for the possibility that brain region affected the results, a one‐way repeated measures ANOVA was performed after controlling for region (motor vs. non‐motor). The main effect of species remained significant, *F* (6, 66) = 4.20, *p* < 0.002.

## 4 DISCUSSION

Using brain tissue from five cetacean species and a rodent control, the current study quantifies the species and layer‐specific features of astrocytes. To date, there is limited knowledge of cetacean glia, and thus potentially limited insight into their novel neurobiology. This study addressed this knowledge gap with three major observations about cetacean astrocytes: 1. the highest distributions were observed in L1 and WM, 2. layer‐specific effects are larger than species‐specific effects, and 3. cell body diameter differs greatly between species. Taken together these results provide valuable insight into the distribution and morphology of cetacean astrocytes.

Results of this study indicate that astrocyte distributions across cortical layers may differ from previously observed distribution patterns for neurons. Among cetacean brains, L1 displayed lower neuron densities than layers 2–5 (Butti et al., 2014; Cozzi et al., 2016; Hof & Van der Gucht, 2007; Ridgway et al., 2019). In contrast with neurons, astrocyte labeling was highest in L1 and WM, with relatively lower levels in layers 2–5. GFAP+ labeling ranging from 3% to 9% in L1 and WM and 0.5% to 4% in L2–5 are consistent with at least one other study of cetacean astrocytes (Vacher et al., 2023). Although both studies focused on mostly adult cetaceans, Garey et al. suggests that age may not affect glial distributions (1986). Due to astrocytes' critical role in regulating neurotransmitter levels and A β protein accumulation (Kim et al., 2024; Rodríguez‐Giraldo et al., 2022), lower astrocytes densities combined with higher neuronal densities in L2‐5 may underlie the observation of Alzheimer's Disease‐like (AD) neuropathology in cetacean brains (Sacchini et al., 2020; Venn‐Watson & Jensen, 2025). Lastly, results in *Kb*, which showed higher levels of L1 astrocytes in the parahippocampal cortex compared to the somatosensory cortex, may indicate regional differences in astrocyte distributions, implications of which require further investigation.

Previous studies have noted high variability in neuronal density between cetacean brains (Haug, 1987; Hof & Van der Gucht, 2007; Ridgway et al., 2019); thus, the current study investigated if the same is true for astrocytes. Although a significant effect of species was observed for astrocyte distributions, the effect of layers was larger. This may indicate that the inter‐individual variability in cetacean brains was actually larger than the intra‐individual variability, although both were highly significant (*p* < 0.003). Another study which examined motor cortex in three dolphin species (Furutani, 2008; bottlenose, Risso's and striped) observed that the laminar patterns of neurons in the cerebral cortex did not differ between animals, which may indicate that differences observed in this study may be driven by the inclusion of both whales and dolphins in the same study. Indeed, in this study the largest differences in astrocyte staining were between the bottlenose dolphin (*Tt*) and the three whale species (Cuvier's, orca and pygmy sperm; *Zc*, *Oo*, *Kb*). To account for this, future studies may consider a repeated‐measures design for each cortical layer to reduce the impact of inter‐individual variability for cell distributions.

Lastly, the difference in astrocyte size has been noted across mammalian species (Verkhratsky & Nedergaard, 2016) and may contribute to differences in intelligence and AD susceptibility. To our knowledge, this is the first study in cetaceans to both measure astrocyte (cell body) diameter and perform rigorous quantitative comparisons between cetacean species and a rodent control. Although cetacean astrocytes did not significantly differ in size compared to rodent astrocytes (*p* > 0.50), differences were observed within cetaceans. The largest difference in astrocyte size was between the false killer (*Pc*) and pygmy sperm (*Kb*) whales. Previous work from our lab (Ridgway et al., 2019) showed astrocyte cell bodies in gray matter ranged from 6.5–7.7 μm in diameter and neurons were slightly larger, 9.9–12.9 μm (although *Kb* and *Pc* were not included in that study). The current study adds to this by demonstrating that WM astrocytes are smallest in *Kb* and range from 2.3–17.6 μm, falling slightly outside the size ranges of gray matter astrocytes. Although previous work from our group had found qualitative associations between neuron density and diving depth (Ridgway et al., 2019), this association does not seem to hold for white matter astrocytes as both *Pc* and *Kb* are deep divers. Instead, overall body size (and brain size) may explain differences between the animals given that the false killer whale is often twice the size of the pygmy sperm whale. Relationships between cetacean neurobiology and other phenotypes such as body size need further exploration as we try to better understand the factors that affect mammalian astroglia.

**FIGURE 4:**
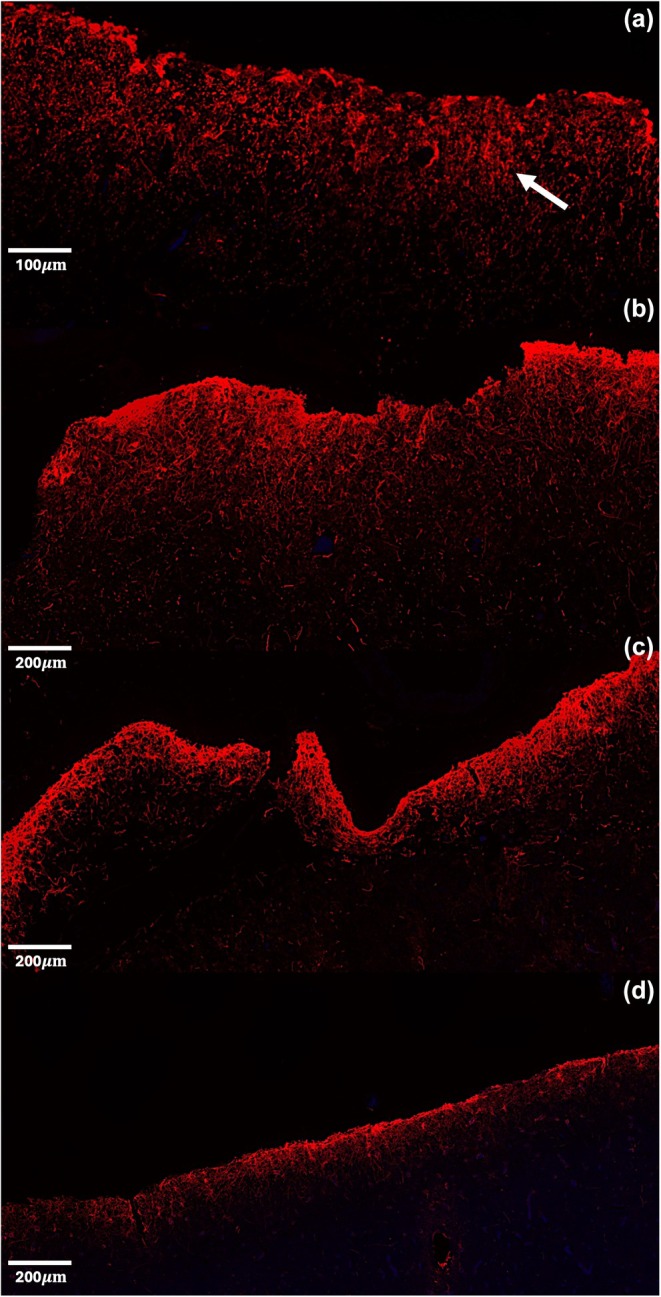
Representative images of anti‐glial fibrillary acidic protein labeling in layer 1. (a) Motor cortex in *Orcinus orca*, (b) motor cortex in *Kogia breviceps*, (c) parahippocampal cortex in *Kogia breviceps*, (d) somatosensory cortex in *Kogia breviceps*. Arrow indicates staining in layer 1.

**FIGURE 5:**
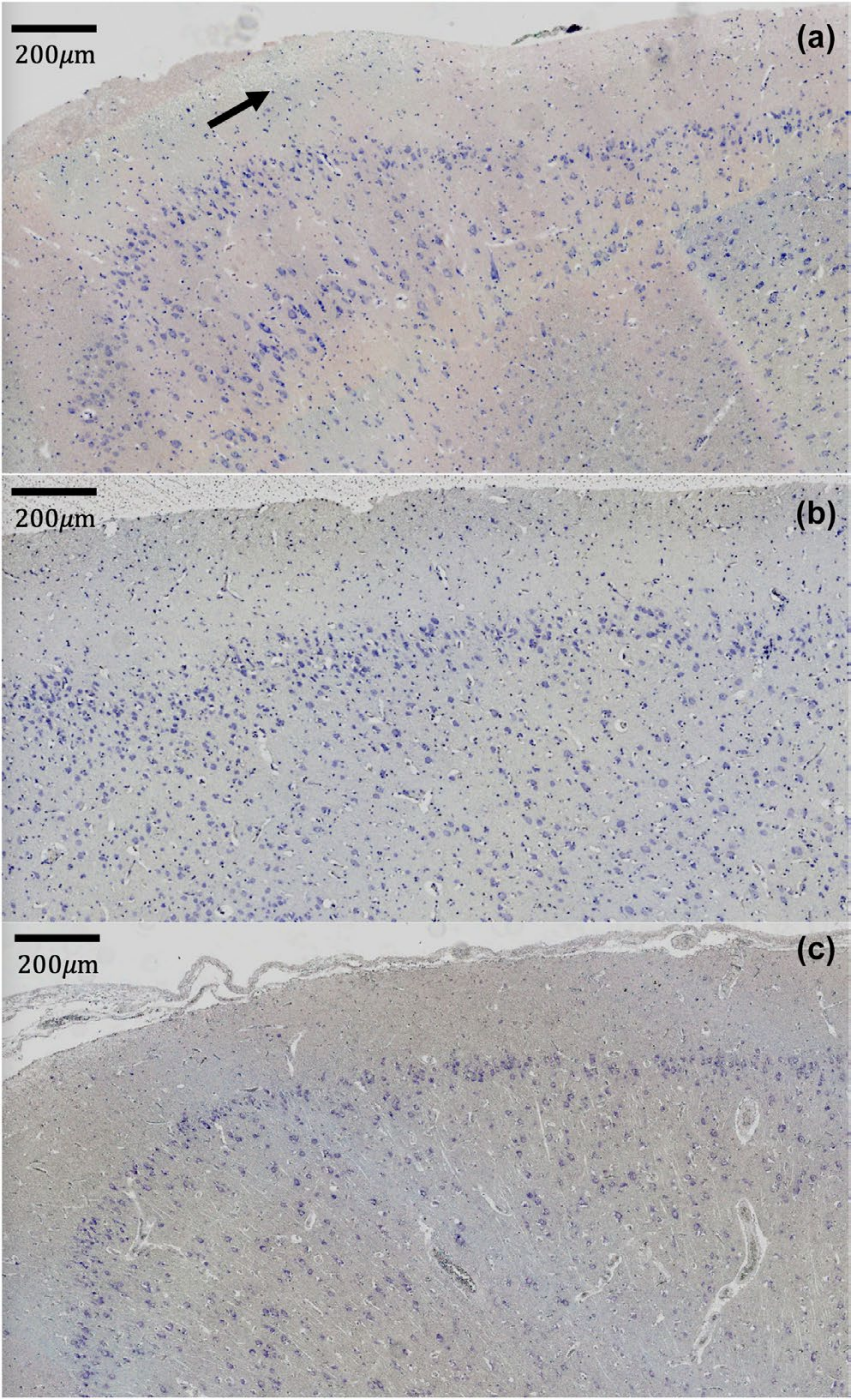
Representative images of Nissl‐stained tissues. (a) Motor cortex in *Orcinus orca*, (b) motor cortex in *Tursiops truncatus*, (c) motor cortex in *Pseudorca crassidens*. Arrow indicates layer 1.

Using relatively rare brain tissue from five cetacean species, this study profiled astrocyte distribution and size. The results were well aligned with previous research; for example astroglia in cetaceans cluster around capillaries in WM as observed in Glezer et al. (1987) and expression patterns of GFAP+ cells matched Vacher et al. (2023). Previous research from our lab (Ridgway et al., 2019) showed that glia density (48,944–29,188 cells/layer) was substantially greater than neuron density (2970–285 cells/layer) in layer 1, across species. This is consistent with the pattern of higher GFAP staining in L1 and lower Nissl staining observed across brain regions (e.g., somatosensory, parahippocampal, auditory cortices; see arrows in Figures 4 and 5) and species. The reverse pattern is observed in L2–5, with lower GFAP and higher Nissl staining, (see Figures 4 and 5). Overall, Ridgway et al. (2019) showed that neuron densities showed greater variability between layers and species than glial densities. Future work in marine mammals should be aware of these species‐ and layer‐ differences in cell distributions.

There are several limitations in this study including the labeling assays and tissue availability. It has been noted that GFAP does not stain all astrocytes (Kimelberg, 2004; Mishima & Hirase, 2010), the number of GFAP negative astrocytes ranges anywhere from 10% in cultured astrocytes (Du et al., 2010) to 40% in rodent hippocampus (Walz & Lang, 1998). Despite this, GFAP labeling is a widely used marker of astrocytes in the brain (Preston et al., 2019) with the caveat that measures of astrocyte density may be underestimated. Tissue availability and quality have also been noted as a challenge in cetacean research (Hunt et al., 2013; Jin et al., 2023) and as evidenced by the limited number of animals in the present study. Tissue was only available from one animal of each species at the time of the analysis. Despite these challenges, this study provides neuroanatomical information of cetacean astrocytes and may provide context to future studies examining cetacean astroglia form and function.

## CONFLICT OF INTEREST STATEMENT

The authors declare no competing interests.

## ETHICS STATEMENT

Animal brains were collected under the authority codified in U.S. Code, Title 10, Section 7524. Secretary of Navy Instruction 3900.41H with AAALAC International accreditation and adheres to the national standards of the U.S. Public Health Service policy on the Humane Care and Use of Laboratory Animals and the Animal Welfare Act.

## Data Availability

Data will be made available upon request via email to corresponding author.
